# Sleep disturbances in Lewy body dementia: A systematic review

**DOI:** 10.1002/gps.5814

**Published:** 2022-09-27

**Authors:** Greg J. Elder, Alpar S. Lazar, Pam Alfonso‐Miller, John‐Paul Taylor

**Affiliations:** ^1^ Northumbria Sleep Research Department of Psychology Faculty of Health and Life Sciences Northumbria University Newcastle upon Tyne UK; ^2^ Sleep and Brain Research Unit Faculty of Medicine and Health Sciences University of East Anglia Norwich UK; ^3^ Translational and Clinical Research Institute Newcastle University Campus for Ageing and Vitality Newcastle Upon Tyne UK

**Keywords:** Lewy body dementia, dementia with Lewy bodies, Parkinson’s disease dementia, sleep, sleepiness, sleep disturbances

## Abstract

**Background:**

Lewy body dementia (LBD) refers to both dementia with Lewy bodies (DLB) and Parkinson's disease with dementia (PDD). Sleep disturbances are common in LBD, and can include poor sleep quality, excessive daytime sleepiness (EDS), and rapid eye movement behaviour disorder (RBD). Despite the high clinical prevalence of sleep disturbances in LBD, they are under‐studied relative to other dementias. The aim of the present systematic review was to examine the nature of sleep disturbances in LBD, summarise the effect of treatment studies upon sleep, and highlight specific and necessary directions for future research.

**Methods:**

Published studies in English were located by searching PubMED and PSYCArticles databases (until 10 June 2022). The search protocol was pre‐registered in PROSPERO (CRD42021293490) and performed in accordance with PRISMA guidelines.

**Results:**

Following full‐text review, a final total of 70 articles were included. These included 20 studies focussing on subjective sleep, 14 on RBD, 8 on EDS, 7 on objective sleep, and 1 on circadian rhythms. The majority of the 18 treatment studies used pharmacological interventions (*n* = 12), had an open‐label design (*n* = 8), and were of low‐to‐moderate quality. Most studies (*n* = 55) included only patients with DLB. Due to the heterogeneity of the studies, we reported a narrative synthesis without meta‐analysis.

**Conclusions:**

At least one form of sleep disturbance may be present in as many as 90% of people with LBD. Subjectively poor sleep quality, excessive daytime sleepiness, and RBD are more common and severe in LBD relative to other dementias.

## Key points

- At least one form of sleep disturbance may be present in as many as 90% of people with Lewy body dementia(LBD).
- Subjectively poor sleep quality, excessive daytime sleepiness, and rapid eye movement behaviour disorder are more common and severe in Lewy body dementia relative to other dementias.
- High‐quality Lewy body dementia‐specific treatment studies are urgently needed for these symptoms, as the current evidence base is very weak.
- Current clinical management guidelines for LBD sleep disturbances mainly include the use of sleep hygiene, or pharmacological agents; some agents are associated with negative side effects.

## 1 INTRODUCTION

Lewy body dementia (LBD) refers to dementia with Lewy bodies (DLB) and Parkinson's disease dementia (PDD), which have an overlapping symptom profile, neuropathology, and treatment response.^1^, ^2^, ^3^, ^4^ DLB is the second most common cause of neurodegenerative dementia (comprising 15%–20% of all dementia cases) after Alzheimer's disease (AD),^5^ and up to 80% of individuals with Parkinson's disease (PD) develop dementia.^6^ The distinction between DLB and PDD is in the timing of symptom onset: DLB is diagnosed when cognitive impairment occurs prior to, or within 1 year of parkinsonism; PDD is diagnosed when cognitive impairment occurs alongside PD.^4^

A distinctive LBD clinical feature is sleep disturbances^7^ and these include poor subjective sleep quality, excessive daytime sleepiness (EDS), and rapid eye movement (REM) behaviour disorder (RBD), These are common: for instance, EDS occurs in approximately 80% of DLB patients.^8^ This is clinically problematic, as patient sleep disturbances can result in increased caregiver distress.^9^ Given the direct link between sleep, health, and cognition, patient sleep disturbances may exacerbate negative physical/psychological health outcomes, and dramatically increase the risk of subsequent cognitive decline.^10^, ^11^ However, despite the high clinical prevalence of LBD sleep disturbances, their aetiology is poorly understood compared to other dementias.^12^

Sleep is a complex process and is assessed and quantified using multiple subjective and/or objective measurement methods.^13^ Objective measures of sleep can include polysomnography (PSG), which is the most accurate method as simultaneous physiological and brain activity are used to accurately classify different stages of sleep^13^ and actigraphy, which uses watches containing accelerometers to estimate sleep and wake based on movement.^14^ Subjective sleep parameters (e.g. sleep quality, duration or experience) can be assessed using questionnaires or sleep diaries.^13^ Sleepiness can be measured subjectively, using self‐report or informant questionnaires, or objectively, using the ‘gold standard’ multiple sleep latency test (MSLT), where PSG is used to quantify sleepiness during a nap opportunity.^15^

The aim of the present review was to firstly, to examine the nature and potential underlying mechanisms of LBD‐specific sleep disturbances; secondly, to summarise treatment studies targeting sleep improvements; thirdly, to highlight specific research directions in terms of aetiology and clinical management.

## 2 METHODS

The search protocol was pre‐registered in PROSPERO (CRD42021293490) and performed in accordance with PRISMA guidelines.^16^

### 2.1 Search strategy

Studies were located by searching PubMED (until 10 June 2022) and PSYCArticles (from 1967 until 10 June 2022) databases using the terms (“dementia with Lewy bodies” OR “dementia with Lewy*” OR “Lewy*” OR “Parkinson's disease dementia” OR “Parkinson's disease with dementia” AND (*“sleep” OR “sleep quality” OR “sleep disorder” OR “sleep disturbances” OR “sleep deficit” OR “sleep impairment” OR “RBD” OR “REM behavior disorder” OR “REM behaviour disorder” OR “rapid eye movement behaviour disorder” OR “rapid eye movement behavior disorder” OR “insomnia” OR “parasomnia” OR “restless legs syndrome” OR “Willis‐Ekbom disorder” OR “periodic limb movement” OR “periodic limb movement syndrome” OR “periodic limb movement disorder” OR “nocturia” OR “sleep apnoea” OR “sleep disordered breathing” OR “obstructive sleep apnoea” OR “central sleep apnoea syndrome” OR “circadian rhythm” OR “circadian rhythm sleep disorder” OR “excessive daytime sleepiness” OR “hypersomnolence” OR “somnolence” OR “sleepiness”*)).

### 2.2 Eligibility criteria

Studies were eligible if participants had DLB/PDD, diagnosed in line with recognised criteria (or previous versions of this diagnostic criteria).^2^, ^17^ Studies were included if they: (a) focussed on the prevalence, aetiology and/or treatment of sleep disturbances; (b) compared subjective/objective sleep between DLB and/or PDD, non‐LBD neurodegenerative dementias, and/or healthy non‐dementia groups; (c) examined associations between sleep and behavioural/neuropsychiatric symptoms; (d) examined if sleep/sleep disorders predicted LBD diagnosis; e) assessed if sleep distinguished LBD from other neurodegenerative dementias or were; (f) were treatment studies with the primary/secondary aim of improving any aspect of sleep or sleepiness (including case study/clinical trial designs). Unpublished studies and pre‐print articles were not sought, but were considered if relevant or referenced in eligible studies.

Identified articles were excluded if they were: (a) duplicate; (b) review articles; (c) non‐English; (d) concerned with non‐LBD dementias, mild cognitive impairment, or individuals without dementia; (e) in abstract format; (f) opinion‐based letters; (g) neuropathological studies, unless relevant to sleep; (h) RBD studies assessing non‐LBD dementia conversion, or (i) animal studies.

### 2.3 Data extraction

Eligible articles were exported to EndNote X9.3 (Clarivate, London, UK) and abstracts were screened. The methodological quality of studies, in relation to their main aims, were rated from 0 to 5 (representing the highest methodological quality) using the Mixed Methods Appraisal Tool (MMAT^18^ ^,^ ^19^). The first 10% of identified papers and MMAT evaluations were checked by another member of the study team (PA‐M) to verify the search strategy.

### 2.4 Narrative synthesis

Due to the heterogeneity of studies and methodological approaches (Table 1), these data were not amenable to meta‐analysis. Therefore, a narrative description is provided, in line with Synthesis without Meta‐analysis (SWiM;^20^) guidelines.

**TABLE 1:** Main focus of Lewy body dementia (LBD) sleep studies and primary sleep measurement tool

Main focus of study	Primary sleep measurement method (excluding treatment studies)
• Subjective sleep (*n* = 20)	Subjective sleep:
• Objective sleep (*n* = 6)	• Participant, caregiver or informant questionnaires (*n* = 12)
• Excessive daytime sleepiness (*n* = 8)	• Clinical evaluations (*n* = 2)
• RBD (*n* = 14)	• Retrospective review of clinical records (*n* = 1)
• Circadian rhythms (*n* = 1)	• Patient, caregiver or family reports (*n* = 1)
• Periodic limb movements, restless legs syndrome or sleep‐disordered breathing (*n* = 3)	• Patient estimated sleep duration (*n* = 1)
	Objective sleep:
• Treatment studies (*n* = 18)	• In‐laboratory video PSG (*n* = 5)
○ Pharmacological (*n* = 12)	○ Examination of retrospective PSG records (*n* = 3)
▪ Donepezil (*n* = 4)	○ Routine clinical visits (*n* = 1)
▪ Rivastigmine (*n* = 1)	○ Comparison to PD patients (*n* = 1)
▪ Melatonin (*n* = 1)	• Actigraphy (*n* = 1)
▪ Galantamine (*n* = 1)	Sleepiness:
▪ Gabapentin (*n* = 1)	• Subjective measurement (*n* = 4)
▪ Armodafinil (*n* = 1)	• Subjective questionnaires (*n* = 4)
▪ Memantine (*n* = 1)	• Objective measurement of hypocretin‐1 (orexin) (*n* = 5)
▪ Clonazepam (*n* = 1)	• Neuropathology (*n* = 2)
▪ Levodopa (*n* = 1)	• Overnight PSG and MSLT (*n* = 2)
○ Non‐pharmacological (*n* = 6)	REM behaviour disorder:
▪ Bright light therapy (*n* = 2)	• Number of iRBD patients who developed LBD at follow‐up (*n* = 8)
▪ Yokukansan (*n* = 2)	• Differences in clinical symptoms, neuropathology or brain metabolism (*n* = 2)
▪ Deep brain stimulation (*n* = 1)	• Comparison of sleep‐enactment behaviours between LBD and PD (*n* = 1)
▪ Yokukansankachimpihange (*n* = 1)	• Retrospective prevalence study (*n* = 1)
	• Association between subjective RBD and hypocretin‐1 (*n* = 1)
	• Neuropathology (*n* = 1)
	Circadian rhythms:
	• Core body temperature and PSG (*n* = 1)
	Periodic limb movements, restless legs syndrome or sleep‐disordered breathing
	• Clinical evaluations and subjective measurements (*n* = 3)
	• PSG (*n* = 2)

Abbreviations: iRBD, idiopathic rapid eye movement behaviour disorder; LBD, Lewy body dementia; MSLT, multiple sleep latency test; PD, Parkinson's disease.

The studies were grouped as follows: (1) subjective sleep: where the primary aim of the study was to investigate the prevalence or nature of LBD subjective sleep alterations; (2) objective sleep; (3) EDS; (4) RBD, where the aim was to assess the frequency or prevalence in LBD and/or quantify the later conversion to LBD; (5) circadian rhythms, where these were assessed subjectively or objectively (i.e., measuring actigraphy, core body temperature, or other circadian rhythm markers); (6) other sleep symptoms, including obstructive sleep apnoea, sleep‐disordered breathing or restless legs syndrome/periodic limb movements (RLS/PLMs); and (7) treatment studies.

## 3 RESULTS

**FIGURE 1:**
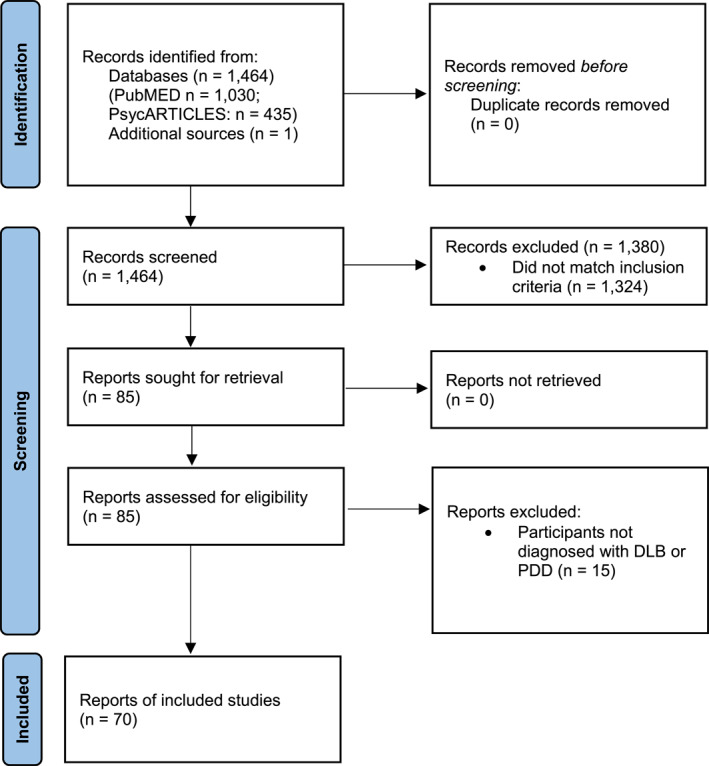
Article selection flowchart

A total of 1464 potentially relevant articles were identified and screened, and one additional result was identified manually. Following screening, 85 articles were chosen for full‐text review. Following review, a final total of 70 articles were included (Figure 1).

### 3.1 Characteristics of studies

There was a large amount of heterogeneity regarding the main study focus and sleep methodologies employed (Table 1). The included studies (Tables 2 and 3) were conducted between 2000 and 2022. The number of participants ranged from one (single‐case studies) to 4531. Fifty‐six studies recruited only DLB,^12^, ^21^, ^22^, ^23^, ^24^, ^25^, ^26^, ^27^, ^28^, ^29^, ^30^, ^31^, ^32^, ^33^, ^34^, ^35^, ^36^, ^37^, ^38^, ^39^, ^40^, ^41^, ^42^, ^43^, ^44^, ^45^, ^46^, ^47^, ^48^, ^49^, ^50^, ^51^, ^52^, ^53^, ^54^, ^55^, ^56^, ^57^, ^58^, ^59^, ^60^, ^61^, ^62^, ^63^, ^64^, ^65^, ^66^, ^67^, ^68^, ^69^, ^70^, ^71^, ^72^, ^73^, ^74^, ^75^ 12 studies recruited both people with DLB and PDD,^76^, ^77^, ^78^, ^79^, ^80^, ^81^, ^82^, ^83^, ^84^, ^85^, ^86^, ^87^ and two recruited only PDD patients^88^, ^89^; these figures include studies that investigated the conversion from idiopathic RBD (iRBD) to DLB or PDD.

**TABLE 2:** Summary of Lewy body dementia (LBD) sleep studies

Author (year)	Country	Study design	Sample size	LBD group	Comparator group	Age (LBD: Mean ± SD)	Sleep measures	Main results	Quality
Subjective sleep									
Boddy et al (2007)^76^	UK	Observational	205	DLB (*n* = 41); PDD (*n* = 24)	AD (*n* = 42); control (*n* = 41); PD (*n* = 39)	DLB: 76 (7); PDD: 73 (6)	ESS, PSQI	PSQI higher in PD, PDD and DLB compared to AD/controls. EDS (ESS >10) more common in PD, PDD and DLB than AD. PDD motor symptomassociated with worse ESS scores.	4
Bli‐wise et al (2011)^21^	USA	Retrospective data analysis	4531	DLB (*n* = 339)	AD (*n* = 4192)	74.0 (7.8)	NPI (night‐time behaviour item)	Sleep disturbances were more frequent in DLB than AD	4
Cagnin et al (2017)^22^	Italy	Observational	62	DLB (*n* = 30)	AD (*n*= 32), control (*n* = 33)	77.52 (4.89)	PSQI, RBD1Q, ESS and 12‐day sleep diaries	Higher ESS scores, and greater frequency of EDS, in DLB compared to AD and controls. Higher PSQI scores in DLB compared to AD, but not controls. Higher frequency of RBD in DLB compared to AD. Sleep diaries: TST and TIB higher in DLB compared to controls, but not AD. NWAK, WASO, SOL and SE were comparable DLB, AD and controls	3
Chwiszczuk et al (2016)^23^	Norway	Cross‐sectional	221	DLB (*n* = 83)	AD (*n* = 138)	77.0 (7.2)	Insomnia (NPI item 11) and MSQ (RBD, PLMS, OSA, SRLC, SW and RLS)	Higher NPI sleep scores in DLB than AD. 73% of DLB participants reported ≥1 sleep problem versus 46% AD. Higher frequency of all sleep problems in DLB compared to AD	4
De‐Oliveira et al (2020)^77^	Brazil	Cross‐sectional	51	DLB (*n* = 37); PDD (n = 14)	N/A	77.76 (7.8)	Estimated daily length of sleep	DLB TST greater than PDD (9.62 vs. 8.07h)	3
Elder et al (2016)^12^	UK	Cross‐sectional	32	DLB (*n* = 32)	N/A	76.16 (7.03)	ESS and PSQI	Self‐reported subjective depression positively associated with ESS and PSQI. ESS and PSQI not associated	4
Farina et al (2009)^24^	Italy	Retrospective and prospective	102	DLB (*n* = 82; probable); DLB (*n* = 20 possible)	N/A	N/A	Clinical evaluation	Sleep disorders, insomnia, EDS, RBD, and confusion upon awakening present in 44%, 26%, 11%, 13% and 5% of patients	1
Galvin et al (2006)^78^	USA	Retrospective and neuropathological	103	DLB (*n* = 20)	AD (*n* = 31); control (*n* = 10); PD (*n* = 42)	71.4 (8.5)	Clinical evaluation	DLB (but not PDD) had a greater frequency of sleep disturbances compared to AD. Sleep disturbances predictive of lewy body pathology	3
Galvin et al (2021)^25^	USA	Cross‐sectional	342	DLB (*n* = 110)	AD (*n* = 78); control (*n* = 53); MCI (*n* = 101)	77.7 (7.6)	NPI, MSQ and SCOPA‐sleep	NPI night‐time behaviours, EDS, RBD symptoms, snorting and choking during sleep more common in DLB	4
Grace et al (2000)^26^	UK	Cross‐sectional	37	DLB (*n* = 17)	AD (*n* = 20)	74.9 (N/A)	ESS, PSQI	Higher ESS and PSQI in DLB compared to AD. More DLB than AD carers reported sleep disturbance‐related distress	4
Guarnieri et al (2012)^79^	Italy	Cross‐sectional	431	DLB and PDD (*n* = 21)	AD (*n* = 204); FTD (*n* = 21); MCI (*n* = 43); VaD (*n* = 25)	78.8 (N/A)	Clinical evaluation, caregiver reports, PSQI, Berlin questionnaire (SDB)	LBD group had the highest frequency (90%) of sleep disturbances: EDS, SDB, insomnia and RBD present in 76%, 71%, 67% and 48% of LBD. No PSQI differences between LBD and other groups	4
Lee et al (2012)^88^	Taiwan	Cross‐sectional	127	PDD (n = 127)	N/A	77.0 (6.3)	NPI	Sleep problems present in 54% of patients. Sleep problems associated with cognition and motor symptoms	4
Mukherjee et al (2017)^27^	India	Cross‐sectional	107	DLB (*n* = 4)	AD (*n* = 66), FTD (*n* = 19), mixed (*n* = 11), VaD (*n* = 7)	63.75 (7.09)	NPI	No difference in NPI between groups	3
Pistacchi et al (2014)^80^	Italy	Cross‐sectional	263	DLB (*n* = 12); PDD (*n* = 19);	AD (*n* = 113); FTD (*n* = 10); MCI (*n* = 27); mixed (*n* = 20);	N/A	Clinical sleep neurology interview, ESS (controls) clinical observation, caregiver information, patient questioning	In DLB and PDD, insomnia (67% and 72%), RLS (83% and 67%), EDS (100% and 83%), RBD (17% and 17%), nightmares (83% and 78%) were present. Only EDS was significantly more common in DLB and PDD relative to other dementias	2
Rongve et al (2010)^81^	Norway and USA	Cross‐sectional	571	LBD (*n* = 39: DLB (*n* = 29) and PDD (*n* = 10))	AD (n = 97); controls (*n* = 420); other (*n* = 15);	78.0 (7.7)	MSQ, ESS and NPI	Sleep problems more common in LBD than AD or other dementias (89% vs. 64% and 73%). RBD and EDS more common in LBD	4
Scharre et al (2016)^82^	USA	Cross‐sectional	63	LBD (*n* = 21: DLB (*n* = 11) and PDD (*n* = 10))	AD (*n* = 21); PD (*n* = 21)	73.95 (4.78)	MSQ, ESS, NPI and caregiver history (sleep apnea)	More EDS, higher prevalence of sleep apnea in LBD than PD or AD, and higher prevalence of RLS than AD	3
Shea et al (2015)^29^	Hong Kong	Cross‐sectional	30	DLB (*n* = 7)	AD (*n* = 14); FTD (*n* = 9)	N/A	Clinical review	RBD more common in DLB	2
Soysal and Tan (2021)^28^	Turkey	Retrospective	82	DLB (*n* = 31)	AD (*n* = 51)	N/A	Insomnia (ISI ≥8) and EDS (ESS ≥11)	Insomnia, but not EDS, more common in DLB (75%) than AD	3
Utsumi et al (2020)^30^	Japan	Retrospective	234	DLB (*n* = 234)	N/A	79.0 (7.5)	Patient, family and caregiver reports.	RBD present in 61% of patients, and was more common in men than women (76% vs. 50%)	4
Van de‐Beek et al (2021)^31^	Netherlands	Prospective	100	DLB (*n* = 73)	MCI (*n* = 27)	69.00 (6.00)	MSQ (RBD)	RBD present in 76%	5
Objective sleep									
Bugalho et al (2019)^32^	Portugal	Retrospective	90	DLB (*n* = 19)	iRBD (*n* = 20); PD (*n* = 51) and age‐normative data	80.21 (8.23)	1 night in‐lab video PSG	Higher SL in DLB than PD. Relative to normative values, DLB showed lower SE%, TST and REM duration; higher SL, WASO& N2 duration	4
Bugalho et al (2021)^33^	Portugal	Retrospective	69	DLB (*n* = 20)	PD (*n* = 49)	80.45 (6.74)	1 night in‐lab video PSG	TST significantly lower in DLB than PD. Mean SE% of 56%, and WASO of 241 min in DLB. Objective sleep did not predict cognitive decline in DLB (at maximum 3.5 years follow‐up).	4
Fernández‐Arcos et al (2019)^47^	Spain	Cross‐sectional	35	DLB (*n* = 35)	N/A	77.7 (6.1)	Clinical assessment and 1 night video PSG	Poor sleep quality (54%) hypersomnia (37%), snoring (60%) and abnormal nocturnal behaviours (77%) reported. Objective SE% low, and high number of aptients with obstructive apneas, PLMs and RBD (50%) of patients). Sleep‐wake architecture abnormalities in 75% of patients: Occipital slowing on wake (34%), absence of sleep spindles/K complexes (13%), slow frequency sleep spindles (13%), delta activity in REM (19%) and REM without atonia (44%)	5
Fukuda et al (2022)^75^	Japan	Observational	18	DLB (*n* = 18)	N/A	78 (7.2)	Objective sleep data, for 1 week, which was measured using a commercially available non‐wearable sensor which is placed on the mattress (Nemuri SCAN)	Sleep data was measured using a commercially available non‐wearable sensor which is placed on the mattress. Median SE% of 68%, median TST of 6.8 h, median number of times the patients got out of bed at night was 3.5 times	2
Kanemoto et al (2020)^34^	Japan	Observational	22	DLB (*n* = 22)	N/A	77.2 (4.4)	NPI and 7 consecutive days of actigraphy (TIB, TST, AAC analysed) alongside caregiver sleep diaries.	No association between actigraphy and NPI sleep. Actigraphy‐measured AAC and TST positively associated with left pulvinar of thalamus FDG uptake. TST positively associated with bilateral orbitofrontal cortex and left thalamus. AAC negatively associated with left thalamus and left parieto‐occipital area. Left pulvinar associated with TST and AAC. TST negatively associated with NPI hallucinations. AAC positively associated with NPI delusions and hallucinations	5
Pao et al (2013)^35^	USA	Retrospective	78	DLB (*n* = 78)	N/A	71 (8)	Clinical video PSG (including split‐night treatment studies)	SE% <80% in 70% of patients. REM sleep without atonia present in 96%. REM sleep not attained in 17%. Approx 75% had arousals not accounted for by movement/breathing disturbance. Primary sleep disorders did not account for poor SE%	4
Terzaghi et al (2013)^36^	Italy	Cross‐sectional	58	DLB (*n* = 29)	PD (*n* = 29)	74.0 (4.9)	1 night video PSG (in‐hospital)	Reduced N1% and increased N2% in DLB relative to PD. Disruptive motor behavioural and confusional events more common in DLB than PD. Mean SE% of 55% and WASO of 183 min in DLB	5
Excessive daytime sleepiness									
Baumann et al (2004)^37^	France, Switzerland and USA	Cross‐sectional	37	DLB (*n* = 10)	AD (*n* = 7); controls (*n* = 20)	72.40 (6.10)	CSF hypocretin‐1 measurement and EDS (DLB *n* = 9)	EDS present in all DLB patients. CSF hypocretin‐1 levels similar between DLB, AD and controls (statistical analysis not conducted)	3
Boeve et al (2019)^38^	USA	Retrospective	159	DLB (*n* = 31)	AD (*n* = 111); bvFTD (*n* = 17)	71.1 (9)	ESS, MSQ	ESS scores higher in DLB than AD and bvFTD. Probable RBD present in 87% of DLB (21% AD and 35% bvFTD).	5
Compta et al (2009)^89^	Spain	Cross‐sectional	63	PDD (*n* = 20)	Controls (*n* = 22); PD (*n* = 21)	72.50 (7.14)	ESS, CSF hypocretin‐1, 1 night video‐PSG and MSLT (in PD (*n* = 8) and PDD (*n* = 7))	Higher EDS scores in PDD than controls, but not than PD. No difference in CSF levels. No difference in MSLT between PD and PDD. Sleep unable to be scored due to altered NREM architecture. Altered NREM and/or slow dominant occipital frequency more common in PDD than PD	3
Ferman et al (2014)^39^	USA	Cross‐sectional	87	DLB (*n* = 61)	AD (*n* = 26)	70.5 (7)	ESS, 1 night PSG, MSLT (DLB *n* = 32)	ESS scores higher in DLB than AD. No PSG differences between DLB and AD. Mean SE% of 72% in DLB. MSLT: DLB patients more likely to have abnormal SL than AD (<10 min: 81% vs. 39%; <5 min: 56% vs. 17%). Mean SL shorter in DLB than AD (6.4 vs. 11.3 min)	5
Kasanuki et al (2018)^40^	USA	Cross‐sectional and neuropathological	60	DLB (*n* = 40)	Control (*n* = 20)	N/A	ESS, MSQ and nBM neuropathology.	EDS present in 58% of DLB patients. Neuronal counts of nBM reduced in DLB compared to controls; DLB EDS + had lower nBM counts than DLB EDS‐ patients. EDS predictive of lower nBM density when adjusted for dementia severity and behavioural symptoms	3
Lessig et al (2010)^41^	USA	Cross‐sectional and neuropathological	43	DLB (*n* = 21)	AD (*n* = 19); control (*n* = 3)	79.5 (7.7)	Unspecified sleep evaluation and neocortical hypocretin levels	Reduced hypocretin fibers and neurons in DLB relative to AD and control. In DLB, cytosolic hypocretin levels and hypersomnolence were positively associated	4
Trotti et al (2021)^42^	USA	Cross‐sectional	126	DLB (*n* = 20)	AD (*n* = 60); controls (*n* = 25); FTD (*n* = 21)	64.9 (9.8)	ESS, NDSQ, hypocretin levels (CSF)	Higher ESS in DLB than controls, but not other dementia groups). CSF hypocretin not different between groups. Nocturia worse in DLB and FTD relative to other groups	5
Yasui et al (2006)^43^	Japan	Cross‐sectional	98	DLB (*n* = 13)	CBD (*n* = 7); PD (*n* = 62); PSP (*n* = 16)	75.7 (6.3)	Medical and family history/clinical evaluation and hypocretin‐1 levels (CSF)	Hypocretin levels not different between groups	4
REM behaviour disorder									
Baumann‐Vogel et al (2020)^44^	Switzerland	Retrospective	671	DLB (*n* = 28)	CBD (*n* = 7); MSA (*n* = 61); PD (*n* = 540); PSP (*n* = 35)	N/A	1 night video PSG	RBD prevalent in 89% of DLB patients	4
Dugger et al (2012)^45^	USA	Cross‐sectional and neuropathological	90	DLB (*n* = 90): DLB RBD+ (*n* = 71) DLB RBD‐ (*n* = 19)	N/A	N/A	MSQ, clinical interview, PSG (n = 34)	DLB RBD+ were predominantly male (82% vs. 47%). DLB RBD + had earlier parkinsonism and visual hallucinations onset, shorter dementia duration than DLB RBD‐. No between‐group difference in pathology	4
Fernández‐Arcos et al (2016)^46^	Spain	Retrospective	203	DLB (*n* = 32)	iRBD (*n* = 134); MSA (*n* = 2); MCI (*n* = 13). PD (*n* = 22);	N/A	Clinical history and assessment and 1 night video PSG	32 patients (16%) diagnosed with RBD at median follow‐up of 5 years	5
Iaccarino et al (2016)^48^	Italy	Retrospective	40	DLB (*n* = 40: DLB RBD+ (n 20); DLB RBD‐ (*n* = 20))	N/A	N/A	RBD1Q, sleep interview and FDG‐PET	DLB RBD + showed decreased metabolism in dorsolateral medial frontal regions, left precuneus, bilateral superior parietal lobule and rolandic operculum, and amygdala, compared to DLB RBD.	3
Inagawa et al (2021)^49^	Japan	Observational	66	DLB (*n* = 19)	AD (*n* = 22); controls (*n* = 25)	81.1 (3.1)	RBDSQ, NPI	Hypocretin‐1 not different between DLB and controls. Hypocretin‐1 positively associated with RBDSQ and NPI (night‐time behaviours and carer distress)	4
							Hypocretin‐1 (CSF)		
Iranzo et al (2006)^83^	Spain	Retrospective	44	DLB (*n* = 6); PDD (*n* = 2)*at follow‐up	N/A	N/A	Video PSG	6 patients (14%) developed DLB and 2 (5%) developed PDD at mean 5.1 year follow‐up	5
Iranzo et al (2013)^50^	Spain	Observational	44	DLB (*n* = 14)*at follow‐up	N/A	N/A	Video PSG	This is an update to study^83^ where patients were followed for an additional 7 years. Of the 44 patients, 32% had developed DLB by 10.5 years follow‐up	5
Iranzo et al (2014)^84^	Spain	Observational	174	DLB (*n* = 29); PDD (*n* = 6)*at follow‐up	N/A	N/A	Video PSG	24 participants (14%) diagnosed with DLB at follow‐up (median 4 years)	5
Miyamoto and Miyamoto (2018)^51^	Japan	Retrospective	273	DLB (*n* = 19)*at follow‐up	N/A	N/A	Video PSG	19 participants (7%) developed DLB at follow‐up (mean period of 4 years)	5
Postuma et al (2009)^52^	Canada	Observational	93	DLB (*n* = 11)*at follow‐up	N/A	N/A	PSG	11 participants (12%) developed DLB at mean follow‐up of 5 years.	5
Ratti et al (2012)^85^	Italy	Cross‐sectional	70	DLB/PDD (*n* = 29)	PD (*n* = 41)	72.3 (6.8)	1 night video PSG	Sleep enactment behaviours consisting of RBD, or occurring upon NREM/REM arousal, more frequent in DLB/PDD than PD. SEBs were related to being male, EDS, higher motor impairment, and lower cognition.	5
Rodrigues Brazète et al (2016)^53^	Canada	Observational	92	DLB (*n* = 14)*at follow‐up	N/A	N/A	Video PSG	14 participants (15%) developed DLB at mean follow‐up of 3.5 years. Higher waking cortical EEG slow‐to‐fast ratio associated with DLB development	4
Schenck et al (2013)^54^	USA	Observational	26	DLB (*n* = 3)	N/A	N/A	Video PSG	3 participants (12%) developed DLB at mean 14.2 years follow‐up.	5
Schmeichel et al (2008)^55^	USA	Cross‐sectional and neuropathological	37	DLB (*n* = 13); DLB RBD+ (*n* = 5)	Control (*n* = 11); MSA (*n* = 13)	77 (2)	Clinical record review and PPT/LDT tegmental nuclei cell count	Reduced PPT/LDT cell count in DLB and MSA relative to controls. PPT/LDT cell loss not associated with RBD presence.	4
Circadian rhythms									
Raupach et al (2020)^56^	Australia	Cross‐sectional	56	DLB (*n* = 6)	Control (*n* = 10); iRBD (*n* = 15); PD (*n* = 31)	68.8 (10.3)	RBDSQ, ESS, SCOPA‐S, 1 night PSG, core body temperature. Circadian variables: Mesor, nadir and amplitude	Higher ESS in DLB than RBD. Compared to controls, CBT amplitude lower in iRBD, PD with REM and DLB. No difference in PSG sleep architecture.	3
Periodic limb movements, restless legs syndrome or sleep‐disordered breathing									
Bhalsing et al (2013)^57^	India	Cross‐sectional	359	DLB (*n* = 5)	Control (*n* = 172); MSA (*n* = 21); PD (*n* = 134); PSP (*n* = 27)	N/A	Clinical evaluation and IRLSSG rating (severity)	RLS: 0% of DLB patients reported RLS.	3
Hibi et al (2012)^58^	Japan	Cross‐sectional	31	DLB (*n* = 9)	AD (*n* = 12); control (*n* = 10)	82.9 (5.9)	1 night PSG (in‐patient)	PLMS: DLB patients had a significantly higher PLMS index than AD. No difference in PSG sleep architecture.	4
Koo et al (2018)^59^	South Korea	Cross‐sectional	92	DLB RBD+ (*n* = 15)	iRBD (*n* = 35); MSA (*n* = 17); PD (*n* = 25)	70.9 (7.5)	1 night PSG	DLB patients had higher PLMS index. No difference in overnight PSG (sleep architecture or sleep apnea variables).	4

Abbreviations: AAC, average activity count, AD, Alzheimer's dementia; bvFTD, behavioural variant frontotemporal dementia; CBD, corticobasal degeneration; CBT, core body temperature; CSF, cerebrospinal fluid; DLB, Dementia with Lewy bodies; ESS, Epworth Sleepiness Scale; EDS, excessive daytime sleepiness; EEG, electroencephalography; FDG, fluorodeoxyglucose; FTD, frontotemporal dementia; iRBD, idiopathic REM Behaviour Disorder; IRLSSG, International Restless Legs Syndrome Study Group; LBD, Lewy body dementia; LDT, laterodorsal tegmentum; MCI, mild cognitive impairment; MSA, multiple system atrophy; MSLT, multiple sleep latency test; MSQ, Mayo Sleep Questionnaire; N/A, not applicable; NPI, Neuropsychiatric Inventory; N2, non rapid eye movement stage 2 sleep; NWAK, number of awakenings; NREM, non‐rapid eye movement sleep; nBM, nucleus basilis of meynert; OSA, obstructive sleep apnea; PET, positron emission tomography; PD, Parkinson's disease; PDD, Parkinson's disease dementia; PSG, polysomnography; PSP, progressive supranuclear palsy; PSQI, Pittsburgh Sleep Quality Index; PPT, pedunculopontine tegmentum; PLMs, periodic limb movements; RBD, rapid eye movement behaviour disorder; RBD1Q, REM Sleep Behavior Disorder; RBDSQ, rapid eye movement behaviour; RLS, restless legs syndrome; SCOPA, Scales for Outcomes in Parkinson's disease; SD, Standard deviation; SE%, sleep eficiency (%); SEB, sleep enactment behaviour; SRLC, sleep‐related leg cramps; SW, sleepwalking; TIB, time in bed; TST, total sleep time; VaD, vascular dementia; WASO, wake after sleep onset.

**TABLE 3:** Summary of Lewy body dementia (LBD) treatment studies

Author (year)	Country	Study design	Intervention	Sample size	LBD group	Other groups	Age (LBD: Mean ± SD)	Primary outcome measure	Sleep measures	*p*‐Value	Effect size	Main results	Quality
Ambar Akkaoui et al (2020)^60^	France	Single case report	Bright light therapy	1	DLB (*n* = 1)	N/A	63 (0)	Not specified	ESS, PSQI, sleep diary	N/A	N/A	Daytime sleepiness and sleep disturbances improved	1
Boeve et al (2003)^61^	USA	Retrospective clinical review	melatonin	14	DLB (*n* = 7)	MCI (*n* = 2); MSA (*n* = 2); narcolepsy (*n* = 2); PD (*n* = 1)	N/A	RBD	N/A	N/A	N/A	No DLB‐specific results provided. Eight patients reported RBD improvements.	0
Edwards et al (2004)^62^	USA	Open‐label study	Galantamine	25	DLB (*n* = 25)	N/A	N/A	NPI‐12 and COGDRAS	PSQI	0.016	N/A	PSQI improvement	3
Fujishiro (2014)^63^	Japan	Single case report	Gabapentin	1	DLB (*n* = 1)	N/A	74 (0)	N/A	N/A	N/A	N/A	RLS improved	2
Grace et al (2000)^26^	UK	Open‐label study	rivastigmine	6	DLB (*n* = 6)	N/A	N/A	ESS, PSQI	ESS, PSQI, individual PSQI items	N/A	N/A	ESS, PSQI, bad dreams, PLMs and confusion upon awakening reduced at 12 weeks	2
Iwasaski et al (2012)^86^	Japan	Open‐label study	Yokukansan	63	DLB/PDD (*n* = 63)	N/A	78.2 (5.8)	Not specified	AD insomnia subscale	0.011	N/A	AD insomnia subscale improved	2
Kazui et al (2017)^64^	Japan	Open‐label study	Donepezil	40	DLB (*n* = 16)	Control (*n* = 24) *used for baseline comparisons only	77.1 (4.6)	Not specified (sleep disturbances)	NPI (sleep disturbances) and actigraphy: AAC, TIB, TST, SL, SnT, WASO, SE%	0.017, 0.044, 0.326, 0.301, 0.679, 0.535, 0.030, 0.196	N/A	NPI improvement and actigraphy improvements in fragmented sleep and AAC	4
Lapid et al (2017)^65^	USA	Open‐label pilot study	Armodafinil	20	DLB (*n* = 20)	N/A	N/A	Efficacy, safety and tolerability study, ESS, MWT	ESS, MWT	<0.001, 0.003	N/A	Improvement in ESS and MWT	4
Larsson et al (2010)^87^	Norway and Sweden	Double‐blind randomised placebo‐controlled trial	Memantine	57	DLB (*n* = 27); PDD (*n* = 30)	N/A	N/A	Clinical global improvement	Probable RBD (SSD) ESS	0.006, 0.552	N/A	Improvement in subjective RBD	3
Maclean et al (2001)^66^	New Zealand	Multiple case reports	Rivastigmine	8	DLB (*n =* 8)	N/A	74.25 (7.36)	None	Subjective clinical reports	N/A	N/A	7 patients reported sleep disturbances. Subjective EDS and nocturnal sleep disturbances improved.	0
Maltête et al (2021)^67^	French	Double‐blind randomised placebo‐controlled crossover trial	Deep brain stimulation	6	DLB (*n =* 6)	N/A	62.2 (7.8)	Safety of DBS	NPI (sleep subscale) and ESS	>0.05	N/A	Subjective reduction in NPI sleep and ESS	3
Manabe (2020)^68^	Japan	Open‐label pilot study	Yokukansanka‐chimpihange	13	DLB (*n* = 5)	DLB pre‐dementia (*n* = 8)	76.45 (7.26)	None stated	RBD: NPI with VAS for frequency and severity	<0.0.01, <0.01, <0.05	N/A	Reductions in NPI, frequency and severity at 4 weeks (overall analysis)	3
Massironi et al (2003)^69^	Italy	Multiple case reports	Clonazepam or donepezil	3	DLB (*n* = 3)	N/A	74.67 (6.51)	RBD	Number of nights with suspected RBD episodes	N/A	N/A	Number of nights with suspected RBD episodes reduced after treatment	1
Molloy et al (2009)^70^	UK	Open‐label pilot study	Levodopa	24	DLB (*n* = 15)	PD (*n* = 9)	76.5 (6.5)	ESS and PDSS	ESS, PDSS and NPI (sleep subscale)	>0.05, >0.003, >0.05	N/A	No improvements at 3 or 6 months	3
Ozaki et al (2012)^71^	Japan	Single case report	Donepezil	1	DLB (*n* = 1)	N/A	80 (0)	Objective sleep spindles (PSG)	Sleep spindles	N/A	N/A	Increase in sleep spindles	2
Sekiguchi et al (2017)^72^	Japan	Open‐label pilot study	Bright light therapy	17	DLB (*n* = 5)	AD (*n* = 8); VaD (*n* = 4)	74.40 (7.33)	Sleep disturbances (NPI‐NH)	NPI‐NH	N/A	N/A	No improvement in DLB	1
Shinno et al (2007)^73^	Japan	Case report	Yi‐Gan San (yokukansan)	1	DLB (*n* = 1)	N/A	81 (0)	None stated	PSG (PLMs)	N/A	N/A	PLMs improved. PSG: TST, SE%, NWAK, N2 and REM duration increased	1
Skjerve and Nygaard (2000)^74^	Norway	Single case report	Donepezil	1	DLB (*n* = 1)	N/A	71 (0)	“Sundowning”: Nocturnal behavioural symptoms	Actigraphy: Daytime and evening activity data	N/A	N/A	Reduction in evening activity and behavioural improvements at 6 weeks	1

Abbreviations: AAC, average activity count per minute (sleep); AD, Alzheimer's dementia; bvFTD, behavioural variant frontotemporal dementia; COGDRAS, Cognitive Drug Research Computerized Assessment System; CBD, corticobasal degeneration; DBS, deep brain stimulation; DLB, Dementia with Lewy bodies; EDS, excessive daytime sleepiness; ESS, Epworth Sleepiness Scale; FTD, frontotemporal dementia; iRBD, idiopathic REM Behaviour Disorder; LBD, Lewy body dementia; MCI, mild cognitive impairment; MSA, multiple system atrophy; MWT, Maintenance of wakefulness test; N/A, not applicable; NPI, Neuropsychiatric Inventory; N2, non rapid eye movement stage 2 sleep; NWAK, number of awakenings; PD, Parkinson's disease; PDD, Parkinson's disease dementia; PDSS, Parkinson's disease sleep scale; PLMs, periodic limb movements; PSG, polysomnography; PSP, progressive supranuclear palsy; PSQI, Pittsburgh Sleep Quality Index; RBD, rapid eye movement behaviour disorder; REM, rapid eye movement sleep; RLS, restless legs syndrome; SD, Standard deviation; SE%, sleep eficiency (%); SNT TST, total sleep time; TIB, time in bed; VaD, vascular dementia; WASO, wake after sleep onset.

Twenty studies investigated subjective sleep,^12^, ^21^, ^22^, ^23^, ^24^, ^25^, ^26^, ^27^, ^28^, ^29^, ^30^, ^31^, ^76^, ^77^, ^78^, ^79^, ^80^, ^81^, ^82^, ^88^ where 12 used participant, caregiver or informant questionnaires^12^, ^21^, ^22^, ^23^, ^25^, ^26^, ^27^, ^28^, ^76^, ^81^, ^82^, ^88^; two relied upon clinical evaluations and participant or informant questionnaires,^79^, ^80^ two relied upon a clinical evaluation^24^, ^78^ and one study each used a retrospective review of clinical records,^29^ patient, family or caregiver reports,^30^ self‐report patient sleep duration estimates^77^ or subjective sleep diaries.^22^

Seven studies focussed on objective sleep^32^, ^33^, ^34^, ^35^, ^36^ ^,^ ^47^ ^,^ ^75^ ^,^ ^81^, where five studies used one night of in‐laboratory video PSG^32^, ^33^, ^35^, ^36^, ^47^; three retrospectively examined PSG records.^32^, ^33^, ^35^ One study examined objective sleep in DLB patients who underwent routine clinical visits^47^ and another compared objective sleep between DLB and PD.^36^ One study used actigraphy, alongside questionnaire and caregiver sleep diaries^34^ and finally, one study measured objective sleep using a non‐wearable commercially available movement sensor, which is placed under a mattress.^75^

Eight studies assessed EDS.^37^, ^38^, ^39^, ^40^, ^41^, ^42^, ^43^, ^89^ One study solely used subjective questionnaire measures.^38^ Four used subjective measures.^38^, ^39^, ^40^, ^42^ Five studies objectively measured cerebrospinal fluid (CSF) levels of the neuropeptide hypocretin‐1 (orexin)^37^, ^41^, ^42^, ^43^, ^89^ and two examined the potential underlying neuropathology of EDS, by obtaining neuronal counts within the nucleus basalis of Meynert (nBM), or measuring neocortical hypocretin.^40^, ^41^ Two used objective measures of sleep and sleepiness (PSG and MSLTs),^39^, ^89^ where only a subset of patients completed MSLTs.

Of the 14 RBD studies,^44^, ^45^, ^46^, ^48^, ^49^, ^50^, ^51^, ^52^, ^53^, ^54^, ^55^, ^83^, ^84^, ^85^ eight examined the number of iRBD patients who were subsequently diagnosed with DLB or PDD.^46^, ^50^, ^51^, ^52^, ^53^, ^54^, ^83^, ^84^ Two assessed differences in clinical symptoms, the underlying neuropathology or brain metabolism, in patients with and without RBD,^45^, ^48^ and one compared sleep‐enactment behaviours (including RBD) between LBD and PD.^85^ One retrospective study examined RBD prevalence in DLB patients who attended a sleep laboratory.^44^ Finally, one study examined associations between subjective RBD and CSF hypocretin‐1^49^ and a further neuropathological study examined the association between DLB cholinergic pedunculopontine and laterodorsal tegmental nuclei (PPT/LDT) counts, and RBD symptom presence.^55^ All but three RBD‐focussed studies used PSG.^48^, ^49^, ^55^

Only one study assessed circadian rhythms,^56^ where DLB core body temperature was measured alongside PSG.^56^ Three studies focussed on PLMs or RLS^57^, ^58^, ^59^ using clinical evaluation and subjective measures,^57^ or objective PSG measures of PLMs.^58^, ^59^ The latter study also measured aspects of sleep‐disordered breathing.^59^

There were 17 specific treatment studies^60^, ^61^, ^62^, ^63^, ^64^, ^65^, ^66^, ^67^, ^68^, ^69^, ^70^, ^71^, ^72^, ^73^, ^74^, ^86^, ^87^ (Table 2); additionally, one open‐label study was conducted as an additional component of a DLB observational study.^26^ Of the 18 treatment studies, 12 used pharmacological interventions,^26^, ^61^, ^62^, ^63^, ^64^, ^65^, ^66^, ^69^, ^70^, ^71^, ^74^, ^87^ including rivastigmine,^26^ melatonin,^61^ galantamine,^62^ gabapentin,^63^ donepezil,^64^, ^69^, ^71^, ^74^ armodafinil,^65^, ^66^ memantine,^87^ clonazepam^69^ and levodopa.^70^ Six used predominantly non‐pharmacological interventions including bright light therapy,^60^, ^72^ deep brain stimulation (DBS) of the nBM,^67^ or the herbal remedies yokukansan^73^, ^86^ and Yokukansankachimpihange.^68^ Most (*n* = 8) treatment studies were open‐label pilot studies^26^, ^62^, ^64^, ^65^, ^68^, ^70^, ^72^, ^86^; 5 were single case reports,^60^, ^63^, ^71^, ^73^, ^74^ two were multiple case reports^66^, ^69^ and one was a retrospective patient clinical review.^61^ There were only two double‐blind randomised controlled trials (RCTs), of memantine and DBS^67^, ^87^; however, sleep was not the primary outcome measure in either: subjective RBD and sleepiness were secondary outcomes in an existing trial of memantine^87^ and the primary aim of the DBS study was to assess the safety and effects upon episodic memory.^67^ The quality of the treatment studies ranged from very poor to moderate (Table 3).

## 4 DISCUSSION

Despite the large heterogeneity of the current studies, LBD is associated with a range of sleep symptoms.

### 4.1 Nature of sleep disturbances

Overall, these results indicate that sleep disturbances are highly prevalent in LBD,^24^, ^79^, ^80^ as despite the heterogeneity in the method of measurement, at least one form of sleep disturbance is present in up to 90% of LBD patients^79^ (Table 4). These results also indicate that subjective sleep disturbances, including poor sleep quality, insomnia, EDS, sleep apnea and RLS, are more frequently observed in LBD than in other dementias, including AD.^21^, ^22^, ^23^, ^25^, ^28^, ^29^, ^30^, ^31^, ^76^, ^79^, ^80^, ^81^, ^82^ LBD sleep disturbances are also more severe, in terms of worse sleep quality and greater daytime sleepiness, relative to other neurodegenerative dementias, including AD.^22^, ^23^, ^26^, ^76^ However, it should be noted that true prevalence studies are needed to confirm these estimates.

**TABLE 4:** Estimated prevalence of common Lewy body dementia (LBD) sleep disturbances (based on cross‐sectional studies)

Symptom	Estimated prevalence	Reference
Excessive daytime sleepiness (subjective)	DLB: 11%–100%	24,80
	PDD: 83%	
Insomnia	DLB: 26%–75%	24,28,80
	PDD: 72%	
Nightmares	DLB: 83%	80
	PDD: 78%	
REM behaviour disorder (subjective)	DLB: 13%–76%	24,30,31,80
	PDD: 17%	
REM behaviour disorder (objective)	DLB: 89%	44
Objective sleep alterations:		
• Low sleep efficiency (sleep quality)	• DLB: 54%–70%	35,36,47
• Abnormal nocturnal behaviours	• DLB: 77%	
• Sleep‐wake architecture abnormalities	• DLB: 75%	

Abbreviations: DLB, dementia with Lewy bodies; LBD, Lewy body dementia; PDD, Parkinsons's disease dementia; REM, rapid eye movement sleep.

It is also highly likely that these sleep disturbances can have a corresponding impact upon the caregivers of people with LBD: in one study, 94% of DLB caregivers reported that patient sleep disturbances were a stressor, compared to 40% of AD caregivers.^26^ Although only three studies directly compared sleep between DLB and PDD groups,^76^, ^77^, ^80^ two indicated that neither subjective sleep quality or EDS differed between DLB and PDD,^76^, ^80^ and one study demonstrated that the estimated nocturnal subjective sleep duration, estimated in the number of hours per day, was greater in DLB compared to PDD (9.62 h compared to 8.07 h).^77^

Rapid eye movement behaviour disorder, both in terms of probable RBD, which has been measured using subjective methods (e.g. questionnaires), or confirmed RBD, which has been diagnosed using PSG, was found to be especially common in DLB and PDD^22^, ^24^, ^25^, ^29^, ^30^, ^31^, ^44^, ^47^, ^79^, ^80^, ^81^, ^85^: for instance, one PSG study showed that approximately 90% of DLB patients demonstrated RBD, which was defined in accordance with current diagnostic criteria.^44^ Although one limitation is undoubtedly that PSG studies may be biased towards patients with more severe RBD symptoms, despite this, the identified subjective and cross‐sectional studies still clearly demonstrate high rates of RBD are apparent in DLB (approximately 80% of individuals).^31^, ^79^ Also of note is that an iRBD diagnosis appears to increase the risk of the subsequent development of DLB or PDD.^46^, ^50^, ^51^, ^52^, ^53^, ^54^, ^83^, ^84^ Whilst a weakness of the studies which longitudinally assess iRBD patients is that they typically rely on small sample sizes and variable follow‐up duration periods, the study with the largest number of iRBD patients who later developed DLB (*n* = 32) found a 16% conversion rate from iRBD to LBD at a median 5‐year follow‐up time point.^46^ The risk of dementia development following an iRBD diagnosis may also increase with time: in one study, 19% of iRBD patients developed DLB or PDD at a mean follow‐up of 5.1 years,^83^ and this rose to 32% by 10.5 years.^50^

Although only five studies have examined objective sleep in DLB using PSG,^32^, ^33^, ^35^, ^36^, ^47^ these are suggestive of clear impairments to objective sleep. All five studies demonstrated that objective sleep efficiency (SE%), which is a marker of sleep quality, was extremely low in DLB; for example, in one study, patients had a mean SE% value of 56%,^33^ which is well below the expected normative values (approx. 80%) for healthy older adults.^90^ High levels of wake after sleep onset (WASO), which indicates the total duration of overnight awakenings, were also apparent from the identified studies.^33^, ^36^ In one study, relative to normative control data, DLB patients showed altered sleep continuity and architecture including lower SE% values, total sleep and REM sleep duration, alongside increases in the time taken to get to sleep, WASO, and stage 2 sleep duration.^32^ Compared to PD, three studies found that people with DLB took longer to get to sleep (SL), and had a lower sleep duration, compared to PD.^32^, ^33^ However, two other studies which have used PSG as a secondary measure, observed no differences in objective sleep between DLB or other groups including AD, PD, iRBD and non‐dementia control groups.^39^, ^56^ However, as impairments to objective sleep are commonly observed in neurodegenerative diseases,^91^ more studies are needed to confirm whether or not if there are clear DLB‐specific alterations in terms of objective sleep continuity or architecture.

Despite the fact that comparatively little work has focussed on objective sleep continuity and architecture, one study showed that sleep architecture alterations were present in 75% of DLB patients, including occipital slowing, delta activity during REM, or the absence of sleep spindles and/or K‐complexes.^47^ These abnormalities were not due to technical failures and prevented the accurate classification of overnight brain activity into different stages of sleep.^47^ Two PSG studies also showed greater levels of PLMs in DLB relative to other neurodegenerative conditions, which can in itself be disruptive to objective sleep.^58^, ^59^ Polysomnography has also been used to demonstrate in the context of a specific test of objective sleepiness (an MSLT), that objectively, DLB patients show greater levels of sleepiness relative to AD,^39^ but that there is no difference between DLB and PD or PDD.^89^ Taken together, these results very speculatively suggest that objective sleep architecture alterations could be apparent in DLB and are worthy of further investigation.

### 4.2 Mechanisms of LBD sleep disturbances

The underlying mechanisms of alterations to sleep, and sleep disturbances are unclear from the identified literature, as only five studies included neuropathological evaluations alongside sleep or sleepiness assessments^40^, ^41^, ^45^, ^55^, ^78^; therefore, there is a clear need for future work to address this specific knowledge gap. Two EDS neuropathology studies conducted neuronal counts of the nBM and assessed neocortical hypocretin levels, respectively.^40^, ^41^ The first study showed that nBM neuronal counts were lower in DLB than in controls and that DLB patients who displayed EDS had lower nBM counts than those without EDS.^40^ The second study found reductions in neocortical hypocretin fibres and neurons in DLB relative to AD and control participants.^41^ Since the nBM has a key role in sleepiness, and the hypocretin/orexin system is wake‐promoting,^92^ speculatively, this could indicate that the presence of EDS in Lewy body dementia is potentially due to LBD‐specific alterations to these key wake‐promoting neurotransmitter systems.

Despite RBD being an extremely common symptom within DLB, the identified literature also showed that the patho‐aetiological investigation of this symptom is currently very limited: in a comparison of DLB patients who exhibited RBD, and those who did not, there were no neuropathological differences between both groups.^45^ One further study found a reduced cell count in the PPT/LDT tegmental nuclei, which are involved in the control of REM sleep, in DLB, relative to controls.^55^ The study concluded that cell losses in this region were not associated with the presence or absence of RBD; however, a major limitation is that very few DLB patients (*n* = 5) had RBD.^55^ Given that cholinergic neurons in the PPT and LDT have a key role in REM sleep control,^92^ this area is certainly worthy of further investigation. Finally, it should be determined if sleep alterations can themselves lead to neuropathological changes: one study observed that the presence of retrospectively evaluated clinical sleep disturbances were predictive of Lewy body neuropathology at autopsy,^78^ although it was not stated which sleep disturbances were predictive of LBD neuropathology.

Several studies have examined the potential biological or neurobiological correlates of sleep and sleepiness, by measuring relevant biological, circadian, or brain markers.^37^, ^42^, ^43^, ^48^, ^49^, ^89^ However, all of the identified studies demonstrate substantial heterogeneity in terms of the experimental methods used and rely on small sample sizes (Table 2). These are discussed in the following section.

Five studies have examined the neurobiological correlates of EDS.^37^, ^42^, ^43^, ^49^, ^89^ These studies examined CSF hypocretin‐1 levels, and found no differences in LBD relative to non‐dementia controls or other neurodegenerative disease groups.^37^, ^42^, ^43^, ^49^, ^89^ Despite the key role of hypocretin‐1 in excessive sleepiness, this would suggest that specific neurobiological alterations may not be the main driver of this common symptom. Intriguingly, one pilot study of circadian measures suggests that alterations to circadian rhythms, which refers to the approximately 24‐h oscillatory rhythms exhibited by the body's various physiological and behavioural processes and which exert a significant influence over the timing and quality of sleep,^93^ might be a feature of DLB.^56^ In this highly‐controlled sleep laboratory study in a small group of patients, reductions were observed in core body temperature amplitude (i.e. where the lowest point of the body temperature has been subtracted from the temperature activity), which was measured from the evening until the following morning, in DLB relative to controls, PD, and RBD groups.^56^

Only two imaging studies have been conducted, where both used 18F‐fluorodeoxyglucose position emission tomography (FDG‐PET). In a study of DLB patients with probable RBD, these patients displayed greater levels of reduced metabolism in a number of regions (e.g. dorsolateral medial frontal, left precuneus, bilateral superior parietal lobule, rolandic operculum and amygdala regions),^48^ which the authors speculated reflects a more severe degeneration of the cholinergic system relative to DLB patients without RBD. In the other imaging study, glucose uptake of the left pulvinar of the thalamus in DLB was positively associated with actigraphically measured total sleep time, and negatively correlated with activity during sleep.^34^ The thalamus has a key role in the control of sleep‐wake cycles,^94^ and as thalamic dysfunction is a feature of DLB, and may have a causative role in the core symptom of cognitive fluctuations,^95^, ^96^, ^97^ the role of the thalamus in DLB‐specific sleep alterations should be explored further.

Finally, several of the identified studies would indicate that sleep, sleepiness, and other LBD related symptoms are associated.^12^, ^49^, ^76^, ^88^ For instance, two studies found that greater subjective excessive daytime sleepiness was associated with worse motor symptoms in PDD^76^ and worse subjective depression in DLB.^12^ Other studies have found associations between sleep problems (assessed using the neuropsychiatric Inventory) and cognition and motor symptoms,^88^ and between subjective sleep quality and subjective depression.^12^ Finally, one study showed that hypocretin‐1 levels were positively associated with subjective RBD, and night‐time sleep disturbance severity, in DLB.^49^ Taken together, this would suggest that there is a complex interplay between sleep, sleepiness and the neuropsychiatric and motor symptom profile of LBD. However, due to the wide heterogeneity in the identified studies, the exact nature of the association between sleep, sleepiness and LBD symptoms are as yet unclear, and this should be investigated further. Well‐designed studies are needed to explore the underlying mechanisms behind these associations, and clarify if there is a direct casual mechanistic link between specific sleep alterations, or sleep disturbances, and particular aspects of the LBD symptom profile, since this may lead to new therapeutic opportunities.

### 4.3 Treatment of LBD sleep disturbances

Despite the clinical relevance and ubiquity of sleep disturbances in LBD, it is perhaps very surprising that few treatment studies have been conducted. In particular, the identified literature demonstrated that there is a dearth of high‐quality RCTs in LBD. Of the 18 treatment studies identified,^26^, ^60^, ^61^, ^62^, ^63^, ^64^, ^65^, ^66^, ^67^, ^68^, ^69^, ^70^, ^71^, ^72^, ^73^, ^74^, ^86^, ^87^ only two were double‐blind RCTs^67^, ^87^; neither of these RCTs had the primary aim of improving sleep or sleep‐related symptoms. Despite the low quality of the identified studies, and the fact that the majority were open‐label pilot studies with small sample sizes,^26^, ^62^, ^64^, ^65^, ^68^, ^70^, ^72^, ^86^ or case reports,^60^, ^63^, ^66^, ^69^, ^71^, ^73^, ^74^ several interventions are of interest and may warrant further investigation, ideally in the context of a sufficiently powered, high‐quality, RCT.

Of the open‐label studies, in terms of DLB pharmacological interventions, the identified results indicated that rivastigmine and galantamine may benefit subjective sleep quality,^26^, ^62^ and that donepezil may improve nocturnal sleep disturbances and fragmented objective sleep.^64^ Rivastigmine and armodafinil were shown to reduce subjective EDS and armodafinil also objectively improved the ability to stay awake.^26^, ^65^ In the only double‐blind RCT of a pharmacological intervention, memantine improved subjective RBD in DLB and PDD, albeit as a secondary outcome measure.^87^ Mechnicanistically, armodfinil is wake‐promoting^65^ and whilst acetylcholine has a role in the complex process of sleep and wake regulation,^98^ future work should investigate whether cholinesterase inhibitors directly benefit sleep, and the mechanistic reasons for doing so, or if the benefit observed to sleep is secondary due to improvements in dementia symptom severity.^26^, ^62^, ^64^

Of the non‐pharmacological interventions, the Japanese herbal intervention yokukansan improved LBD insomnia symptoms, although this was measured using an AD insomnia subscale^86^; similarly, the related yokukansankachimpihange herbal intervention reduced subjective nocturnal disturbance behaviour frequency, although these were open‐label studies.^68^ An open‐label study showed that bright light therapy did not improve DLB sleep disturbances, but did so in AD^72^; however, a single case study in DLB suggested that light therapy improved subjective daytime sleepiness and reduced subjective sleep disturbances.^60^ Finally, deep brain stimulation of the nBM did improve sleep and daytime sleepiness in DLB; however, the primary aim of this study was to examine the safety and efficacy of this procedure upon cognitive outcomes, and there are outstanding concerns regarding the adverse effects of this procedure.^67^ The underlying mechanisms of these interventions are less clear relative to pharmacological interventions, although light is a very important cue for sleep timing and quality,^99^ and nBM stimulation may be beneficial due to this region's key role in regulating sleepiness.^92^

### 4.4 Future research directions

Overall, as these results indicate that there is a very high prevalence of sleep disturbances and EDS in Lewy body dementia, and that they are complex in nature. Importantly, our results indicate that the majority of the studies have focussed on DLB.^12^, ^21^, ^22^, ^23^, ^24^, ^25^, ^26^, ^27^, ^28^, ^29^, ^30^, ^31^, ^32^, ^33^, ^34^, ^35^, ^36^, ^37^, ^38^, ^39^, ^40^, ^41^, ^42^, ^43^, ^44^, ^45^, ^46^, ^47^, ^48^, ^49^, ^50^, ^51^, ^52^, ^53^, ^54^, ^55^, ^56^, ^57^, ^58^, ^59^, ^60^, ^61^, ^62^, ^63^, ^64^, ^65^, ^66^, ^67^, ^68^, ^69^, ^70^, ^71^, ^72^, ^73^, ^74^, ^75^ Whilst we have identified clear knowledge gaps in a range of areas (e.g. whilst there is a high prevalence of subjective and objective sleep disturbances in LBD, their underlying mechanisms are unclear), currently, very little is known if there are specific differences between DLB and PDD in terms of sleep and more comparative studies should investigate this; it is possible that there may be differing symptom profiles and treatment responses with regards to sleep between both groups.

There are a number of other specific future directions. Firstly, high‐quality LBD‐specific treatment studies are urgently needed for these symptoms. From the identified literature, the quality of treatment studies appears to be weak and is primarily limited to poor‐quality open‐label designs with insufficient statistical power. Current clinical management guidelines for LBD sleep disturbances mainly include the use of sleep hygiene, or pharmacological agents such as clonazepam, melatonin and memantine for RBD^100^; however, some agents are associated with negative side effects, which can impact on cognition and cause further sleepiness.^7^ Despite their clinical use, high‐quality RCTs of these agents are yet to be undertaken. This is important because the current evidence base which is used to inform the treatment of sleep disturbances in LBD is very weak, and based on the findings from PD and iRBD, rather than LBD populations.^7^ It is particularly surprising that there were no trials which assessed melatonin in LBD. Melatonin is considered to be a first‐line treatment for symptomatic RBD, and whilst the exact mechanism of action is not well‐understood, it does have a favourable safety profile.^101^ Given the high preponderance of RBD in Lewy body dementia patient groups, this agent is particularly worthy of further investigation. Additionally, given that neuroleptic sensitivity and polypharmacy is an issue in LBD^102^, ^103^ non‐pharmacological behavioural methods including sleep hygiene, exercise or increased ambient light exposure should be trialled; these techniques have certainly shown promise in other neurodegenerative conditions.^104^

Secondly, the exact nature of the potential link between sleep, sleepiness, and LBD behavioural and neuropsychiatric symptoms^12^, ^34^, ^88^ remains to be elucidated. For instance, two studies observed a link between subjective sleep, EDS, and subjective depression, and between objective total sleep time, nocturnal activity, and hallucinations, respectively.^12^, ^34^ It is not clear if LBD directly affects sleep, or EDS, which may in turn, exacerbate particular symptoms; if there is a bi‐directional sleep and symptom link, or a common underlying pathway. For instance, in the case of depressive symptoms and daytime sleepiness, both might be driven by LBD‐specific noradrenergic deficits.^105^, ^106^ This should be examined further as if there is a bi‐directional link between sleep, EDS and behavioural and neuropsychiatric symptoms, then this may provide a therapeutic opportunity: it is possible that by improving sleep quality or timing, this may also improve behavioural and neuropsychiatric symptom severity. Depression is likely to be one such target, as depression is particularly common in DLB, with a prevalence of approximately 60%,^107^ and there is a very strong bi‐directional relationship between sleep, the clinical sleep problem of insomnia disorder, and depression.^108^ As an indication of the potential therapeutic applications in LBD, a recent clinical trial showed that in older adults without dementia, who had comorbid insomnia disorder and depression, the non‐pharmacological treatment of cognitive behavioural therapy for insomnia improved both insomnia and depression severity.^109^

One particular knowledge gap, which is worthy of further investigation, is regarding the potential association between EDS and cognitive fluctuations. Speculatively, this may be due to LBD‐specific alterations to circadian rhythms, as cognitive performance across multiple domains and the underlying neural activity shows a marked circadian modulation.^110^, ^111^, ^112^, ^113^ Typically, the circadian drive for sleep is highest in the early morning hours^114^ and one pilot study has shown that alterations to circadian rhythms may be a feature of DLB.^56^ Clinically, the frequency of cognitive fluctuations varies considerably: cognitive fluctuations have been reported to occur over short periods (e.g. minutes or hours) and longer periods (e.g. daily).^115^, ^116^ Although it is well‐established that the timing and duration of sleep, and sleep deprivation, has a direct impact upon cognitive performance,^117^ the contribution of sleep and sleep disruption to cognitive fluctuations is currently less clear, despite multiple brain regions which are relevant to sleep (e.g. the thalamus) being involved in this symptom.^118^, ^119^ As immediate fluctuations may reflect LBD‐specific impairments to information processing, or transient cortical or subcortical synaptic disturbances,^120^ future work should attempt to clarify the precise impact of sleep upon this poorly understood symptom. Intriguingly, one pilot study of circadian measures suggests that alterations to circadian rhythms might be a feature of DLB: one highly controlled sleep laboratory study in a small group of patients found reductions in core body temperature amplitude (i.e. where the lowest point of the body temperature has been subtracted from the temperature activity), which was measured from the evening until the following morning, in DLB relative to controls, PD, and RBD groups.^56^ Given the rhythmicity of cognitive fluctuations,^115^, ^116^ the potential mechanistic role of circadian rhythm dysregulation, and the circadian interaction with sleep, should be examined further in relation to this core symptom.

Similarly, the natural history of sleep disturbances in LBD is yet to be determined: *post mortem* neuropathological investigations, alongside a detailed longitudinal assessment of subjective and objective sleep, may help delineate the specific pathoaetiological contributions. One particularly important area for future research is to examine the link between subjective sleep quality and continuity, or sleep disturbances, and cognition. The impact of poor sleep upon cognition is well established: multiple domains of cognition, including sustained attention, working memory, and decision‐making, are negatively impacted by insufficient sleep duration and quality.^121^, ^122^, ^123^ Additionally, evidence now increasingly shows that sleep disturbances are mechanistically involved in neurodegeneration.^11^, ^124^

Thirdly, relatively few studies have objectively measured sleep using PSG. Although those studies which have been conducted suggest LBD‐specific disruptions to sleep continuity,^32^, ^33^, ^35^, ^36^, ^47^ there are potential methodological confounders, since a particular issue is that in all studies, sleep was only measured on one night. This is problematic as alterations to objective sleep continuity and architecture are consistently observed during the first night of a PSG study, potentially due to the unfamiliarity of the sleep laboratory environment, and subsequent nights are therefore more representative of normal sleep.^125^, ^126^

Polysomnography is also relevant as objective sleep continuity and architecture is of increasing importance: studies in healthy individuals and AD suggest that disruption to specific stages of sleep influences neurodegeneration.^127^, ^128^ Notably, the link between sleep architecture and cognition has not been examined in LBD; two relevant and specific non‐rapid eye movement sleep (NREM) features are sleep spindles and slow‐wave sleep; both of which reflect thalamocortical network integrity.^129^, ^130^ Sleep spindles are particularly relevant as they originate in the thalamus, and reduced sleep spindles in PD predict subsequent PDD.^129^, ^131^ These NREM measures might influence or reflect cognitive symptom severity: sleep spindles are strongly associated with plasticity, learning and memory.^132^, ^133^, ^134^ One DLB case study found that donepezil increased sleep spindle activity^71^; if NREM measures are associated with cognition, sleep spindle manipulation may also benefit LBD cognition. Additionally, sleep spindles have a protective role against sleep disruption^135^ and an increase in spindles may also improve sleep quality.

Future studies should consider home sleep measurement, which enables sleep information to be collected over longer time periods, as this might provide a greater insight into the impact of sleep upon symptoms or disease progression. For instance, home‐based PSG recording, which has been used to examine sleep architecture in dementia caregivers^136^ may be cheaper and more tolerable for patients than attending a hospital or research sleep laboratory.^137^ Alternative sleep measurement methods might include behind‐the‐ear EEG electrodes, which are easy to apply, less intrusive than PSG, and show a good level of agreement with PSG.^138^ Finally, actigraphy, as a non‐invasive method of measurement, could be used to assess natural sleep/wake patterns over an extended period of time. LBD symptoms may have a circadian element, or be driven by sleep‐related brain regions: cognitive fluctuations are periodic in nature and may have a thalamocortical basis^116^, ^139^; actigraphy may provide an insight into this symptom.

## Data Availability

Data sharing is not applicable to this article as no new data were created or analyzed in this study.
